# Genetic Deletion of *Emp2* Does Not Cause Proteinuric Kidney Disease in Mice

**DOI:** 10.3389/fmed.2019.00189

**Published:** 2019-08-27

**Authors:** Michael D. Donnan, Rizaldy P. Scott, Tuncer Onay, Antoine Tarjus, Ummiye Venus Onay, Susan E. Quaggin

**Affiliations:** Division of Nephrology and Hypertension, Feinberg Cardiovascular and Renal Research Institute, Northwestern University Feinberg School of Medicine, Chicago, IL, United States

**Keywords:** *Emp2*, nephrotic syndrome, proteinuria, smooth muscle, vascular

## Abstract

Nephrotic syndrome is one of the most common glomerular diseases in children and can be classified on the basis of steroid responsiveness. While multiple genetic causes have been discovered for steroid resistant nephrotic syndrome, the genetics of steroid sensitive nephrotic syndrome remains elusive. Mutations in Epithelial Membrane Protein 2 (*EMP2*), a member of the GAS3/PMP22 tetraspan family of proteins, were recently implicated as putative monogenic cause of steroid sensitive nephrotic syndrome. We investigated this hypothesis by developing *Emp2* reporter and knockout mouse models. In *lacZ* reporter mice (engineered to drive expression of the enzyme β-galactosidase under the control of the endogenous murine *Emp2* promoter), *Emp2* promoter activity was not observed in podocytes but was particularly prominent in medium- and large-caliber arterial vessels in the kidney and other tissues where it localizes specifically in vascular smooth muscle cells (vSMCs) but not in the endothelium. Strong *Emp2* expression was also found in non-vascular smooth muscle cells found in other organs like the stomach, bladder, and uterus. Global and podocyte-specific *Emp2* knockout mice were viable and did not develop nephrotic syndrome showing no evidence of abnormal glomerular histology or ultrastructure. Altogether, our results do not support that loss of function of *EMP2* represent a monogenic cause of proteinuric kidney disease. However, the expression pattern of *Emp2* indicates that it may be relevant in smooth muscle function in various organs and tissues including the vasculature.

## Introduction

Nephrotic syndrome (NS) encompasses a triad of clinical features including proteinuria (≥3 g/24 h), low serum albumin (hypoalbuminemia), and edema, and is often accompanied by complications such as secondary hyperlipidemia, thrombotic events, and infections caused by encapsulated organisms (1). Approximately 90% of nephrotic patients respond to corticosteroid treatment and are classified as having idiopathic steroid-sensitive NS (SSNS). The remaining 10% of cases which are refractory to corticosteroids are categorized as steroid-resistant NS (SRNS). Mutations in genes highly expressed in the podocytes have been identified in about two thirds of SRNS patients (2). Many of these SRNS-linked genes encode for proteins that are integral components of the slit diaphragms, regulators of actin cytoskeleton dynamics and cell-matrix interactions, essential for mitochondrial function, and podocyte integrity (3). In contrast, the etiology and pathological mechanism of SSNS remain largely unknown. In ~3% of SSNS patients affected siblings were identified hinting at a possible heritable cause for SSNS (4, 5), although earlier mutation and genome-wide linkage analysis in SSNS-afflicted families fail to uncover a definitive causative gene (6–8). Now, a recent study involving three families suggested that mutations in Epithelial Membrane Protein 2 (*EMP2*) as potential monogenic autosomal recessive causes of childhood onset SSNS (9).

*EMP2* is a member of the growth arrest-specific gene 3/peripheral myelin protein 22 (GAS3/PMP22) family of tetraspan proteins (10–12). Mapped to chromosome 16p13.2, *EMP2* was initially identified by its close homology with *PMP22* (~40% amino acid sequence similarity) (13, 14). *EMP2* translates to an 18 kD protein with four transmembrane domains and multiple consensus sites for N-linked glycosylation in the first hydrophilic domain (13, 15). Tissue distribution and cellular localization of *EMP2* has not been fully characterized before although *EMP2* is known to be prominently expressed in the lungs (10, 14, 16–18). Several cellular functions have been ascribed to *EMP2* including: (1) regulation of cell adhesion involving FAK/Src and β1-integrin; (2) facilitation of trafficking of glycophosphatidylinositol (GPI)-anchored proteins into lipid rafts and regulation of caveolar organization; and, (4) regulation of angiogenesis through induction of *VEGFA* expression (10, 12, 17, 19–21). *EMP2* expression is upregulated in a number of human cancers including endometrial cancers, ovarian cancers, glioblastomas, urinary bladder urothelial carcinoma, and nasopharyngeal carcinomas (10, 14, 22) where it likely plays a role in tumorigenesis through modulation of integrin-dependent cell adhesion (23, 24) and/or promotion of neoplastic angiogenesis (21, 23). *EMP2* has been shown to be important for blastocyst implantation and has been identified as a susceptibility factor for chlamydial infectivity, both likely via its role in regulation of integrin-dependent cell signaling (25–27).

The exact function of *EMP2* in the kidneys remains poorly understood. *EMP2* deficiency has been shown to exacerbate drug-induced injury of zebrafish and cultured human podocytes (28). In this study, we established *Emp2* reporter and conditional mutant alleles in the mouse allowing us to: (1) assess comprehensively the tissue distribution of *Emp2* expression; and (2) address whether loss of function of *Emp2* is a monogenic cause for nephrosis. We conclude that *Emp2* is not highly expressed in glomeruli and that global genetic inactivation of *Emp2* does not affect viability or cause proteinuric kidney disease in mice.

## Results

### Renal Expression of *Emp2*

**Figure 1:**
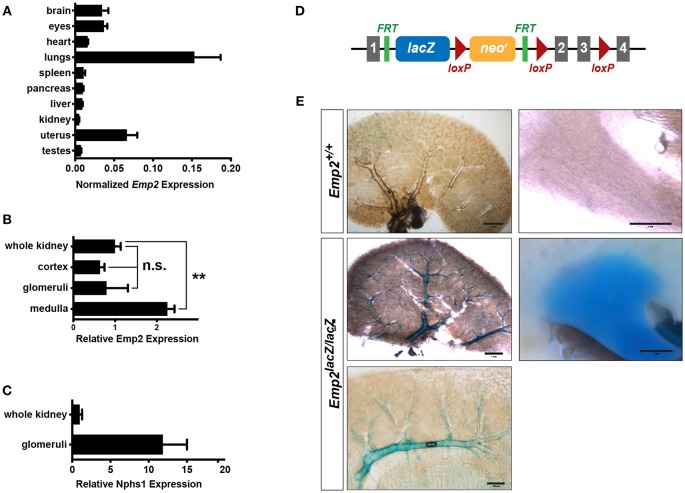
Renal expression of *Emp2*. **(A)** Survey of tissue expression of *Emp2* by qRT-PCR analysis. *Emp2* transcript levels were normalized to that of *Gapdh*. n ≥ 3 animals per group. **(B)** *Emp2* expression is not particularly enriched in glomeruli. Comparison of *Emp2* transcript level normalized to *Gapdh* relative to whole kidney expression in renal cortex, purified glomeruli, and renal medulla. *n* = 3 animal per group. **P < 0.005 **(C)** *Nphs*1 expression is enriched in glomeruli. Comparison of normalized *Nphs1* level between purified glomeruli and whole kidneys. *n* = 3 animal per group. **(D)** Schematic diagram of the *in vivo Emp2* expression reporter construct used in this study. A dual *lacZ*-neomycin resistance cassette was inserted between exons 1 and 2 in the *Emp2* locus to create the *Emp2*^*lacZ*^ reporter allele, where β-galactosidase (β-gal) expression is driven by the endogenous mouse *Emp2* promoter. The conditional floxed allele of *Emp2* (*Emp2*^*flx*^) was subsequently derived from this reporter construct. Gray boxes, *Emp2* exons; green ticks, FRT or Flp recombinase recognition sites; and red triangles, loxP or Cre recombinase recognition sites. **(E)** *Emp2* expression pattern in the kidney. Comparison of *Emp2* promoter activity (based on β-gal histochemical staining) in vibratome slices of kidneys from control (*Emp2*^+/+^) and *Emp2*^*lacZ*^ reporter mice revealing strong *Emp2* expression localized to medium and high-caliber renal vessels and also the renal medulla. Scale bars: 2 mm, middle left panel; 1 mm, upper left/right panel, middle right panel; 200 μm lower panel.

Using quantitative real-time PCR (qRT-PCR) analysis, we surveyed the relative expression of *Emp2* transcript across multiple organs and tissues in mice (Figure 1A). *Emp2* is most strongly expressed in the lungs. High levels of expression were also found in the brain, eyes and the uterus. Modest expression levels were found in the heart, spleen, pancreas, liver, and kidneys. Within the kidney, *Emp2* expression is >2-fold higher in the renal medulla compared to whole kidneys (Figure 1B). *Emp2* expression is not particularly enriched in the renal cortex or isolated glomeruli relative to whole kidneys. In contrast, the transcript level of *Nphs1* (a podocyte-specific gene encoding for nephrin) is ~12-fold higher in isolated glomeruli compared to whole kidneys (Figure 1C).

Tissue localization of *Emp2* expression was analyzed by whole-mount β-galactosidase (β-gal) histochemistry in reporter mice homozygous for the *Emp2*^*lacZ*^ gene trapped allele where prokaryotic β-gal expression is driven by the endogenous *Emp2* promoter (Figure 1D). Control wild-type mice without the *Emp2*^*lacZ*^ allele lack or have negligibly low-levels of background β-gal activity. In 400 μm-thick vibratome sections of the kidney, prominent β-gal activity localizes within large caliber blood vessels (including cortical radial, arcuate, and interlobar vessels), and the renal papilla (Figure 1E). Within the cortex β-gal activity is limited to arcuate vessels and their immediate radial branches but is notably absent in glomeruli and their associated efferent and afferent arterioles. Altogether, these results indicate that in the kidney *Emp2* has minimal expression within podocytes and has a predominantly vascular pattern of expression.

### Vascular Expression of *Emp2*

**Figure 2:**
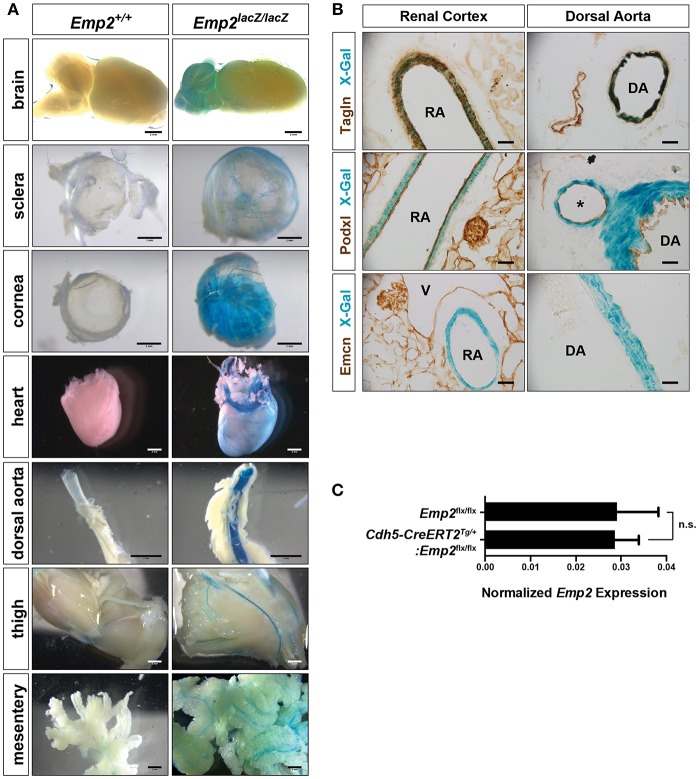
Vascular expression of *Emp2* in multiple tissues. **(A)** β-gal histochemical staining of whole-mount organs and tissues (brain, eye sclera, cornea, heart, dorsal aorta, hind limbs, and mesentery) from control and *Emp2*^*lacZ*^ reporter mice showing vascular pattern of expression. Staining was absent in control mice. Scale bars: 2 mm, **(A)** brain, heart, dorsal aorta, thigh, mesentery; 1 mm, sclera, cornea. **(B)** Vascular expression of *Emp2* is restricted to arterial smooth muscles. In the renal cortex, *Emp2* promoter activity (blue X-gal stain) localizes to Tagln^+^ vascular smooth muscles but not Podxl^+^ arterial endothelium in the renal cortex and dorsal aorta. Emcn^+^ veins are unstained. RA, renal artery; DA, dorsal aorta; V, vein; and *, branching artery. Scale bars: 60 μm. **(C)** *Emp2* expression in the kidney is unchanged upon endothelial-specific deletion of the *Emp2* gene. Normalized *Emp2* expression in kidneys, measured by qRT-PCR analysis, from homozygous *Emp2*^*flx*/*flx*^ animals and tamoxifen-induced endothelial-specific conditional *Emp2* knockout mice (*Cdh5-CreERT2*^*Tg*/+^*:Emp2*^*flx*/*flx*^). *n* = 5 animals per group.

A distinctive vascular pattern of expression of β-gal is found in multiple organs of the *Emp2*^*lacZ*^ reporter mice including the brain, eye sclera, cornea, heart, dorsal aorta, thigh musculature, and gut mesentery (Figure 2A). We cryosectioned thick vibratome slices of kidneys and dorsal aorta stained by X-gal histochemistry so we can determine specific tissue localization of β-gal activity in *Emp2*^*lacZ*^ mice (Figure 2B). In the kidneys, β-gal activity is restricted to high caliber renal arterial vessels where it is particularly located in Tagln^+^ (SM22α^+^) vascular smooth muscle cells (vSMCs) and is absent in Podxl^+^ arterial endothelial cells and renal podocytes. In contrast, Emcn^+^ renal veins, and glomerular endothelial cells completely lack staining for β-gal activity. Likewise, in the dorsal aorta, β-gal activity is confined to the Tagln^+^ vSMCs and is not seen in the Podxl^+^ endothelial compartment. To validate specific cellular localization of *Emp2* expression in the vasculature, we generated tamoxifen-inducible endothelial-specific conditional *Emp2* knock out mice (*Cdh5-CreERT2*^*Tg*/+^*:Emp2*^*flx*/*flx*^). *Emp2* expression within the kidney is unchanged upon endothelial specific knockout of the *Emp2* gene (Figure 2C) consistent with the lack of β-gal activity in the endothelial compartment of *Emp2*^*lacZ*^ reporter mice. We conclude from these results that vSMCs but not their associated endothelial cells account for the vascular pattern of expression of *Emp2*.

### Non-vascular Expression of *Emp2*

**Figure 3:**
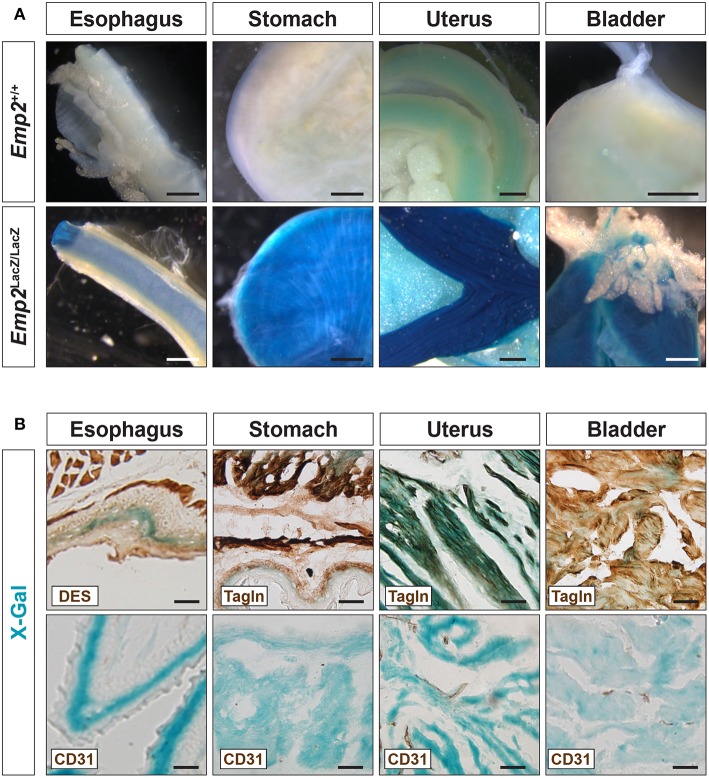
*Emp2* is expressed in smooth muscles. **(A)** *Emp2* is expressed in non-vascular smooth muscles. Strong β-gal histochemical staining of the esophagus, stomach, uterus, and bladder of *Emp2*^*lacZ*^ mice. Scale bars: 1 mm. **(B)** Immunohistochemical staining reveals that the *Emp2* expression is overlaps with Des^+^ (esophagus) and Tagln^+^ (stomach, uterus, and bladder) smooth muscles but not to CD31^+^ endothelia. Scale bars: 200 μm.

In adult mice, pronounced β-gal activity was additionally seen in several smooth muscle tissues including the esophagus, stomach, uterus, and bladder (Figure 3A) but was absent from skeletal muscle tissue such as the thigh (Figure 2A). In cryosections of these tissues, β-gal activity was found primarily in Tagln^+^ or Des^+^ smooth muscle cells (SMCs) and absent in the CD31^+^ or endothelia (Figure 3B). *Emp2* is therefore broadly expressed in different subsets of SMCs both vascular and non-vascular but is absent in skeletal muscle.

**Figure 4:**
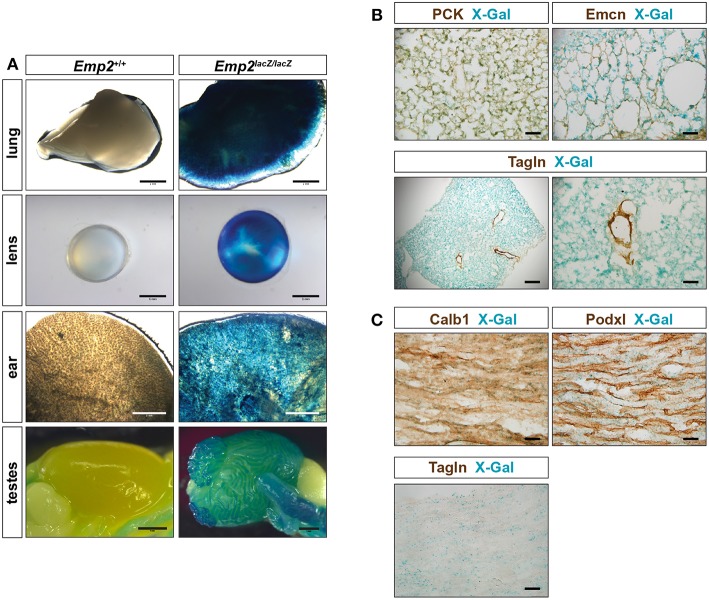
*Emp2* is expressed beyond smooth muscles. **(A)** *Emp2* also has non-smooth muscle expression in other organs. Strong *Emp2* promoter activity in *Emp2*^*lacZ*^ reporter mice in the lungs, eye lens, ear, and testes. Scale bars: 2 mm, lung and ear; 1 mm lens and testes. **(B)** Pulmonary expression of *Emp2* is primarily in pneumocytes. *Emp2* promoter activity (X-gal staining) in the lungs co-localizes with pan-cytokeratin (PCK) immunoreactivity in alveolar epithelial cells (pneumocytes) but not with Emcn (endothelial cells) or smooth muscles (Tagln). Scale bars: 200 μm, lower left; 50 μm remaining. **(C)** Non-vascular expression of *Emp2* in the renal papilla. Prominent *Emp2* promoter activity in the renal papilla co-localizes with Calb1 (principal collecting duct epithelia) and not Podxl (endothelial cells), or Tagln (smooth muscle) immunoreactivities. Scale bars: 50 μm.

*Emp2* expression was additionally observed in non-smooth muscle tissues in several organs. Pronounced β-gal reporter activity was seen in the lungs, eye lens, ear, testes (Figure 4A) and renal papilla (Figure 1E). β-gal activity in the lungs are confined to alveolar pneumocytes that co-stain with antibodies against pan-cytokeratins (PCK) (Figure 4B). β-gal activity is additionally seen in the Tagln^+^ vSMCs of the high caliber arterial vessels within the lung however appears absent from the Tagln^+^ airway smooth muscles. There is no co-localization of Emcn immunoreactivity with β-gal activity in the lungs indicating that *Emp2* expression is negligible or absent in alveolar endothelium.

In the kidney, non-vascular expression of *Emp2* was found primarily in the renal papilla. *Emp2* promoter activity within the renal papilla co-localizes with Calb1 (principal collecting duct epithelia) and not with Podxl (endothelial cells), or Tagln (smooth muscle) immunoreactivities (Figure 4C).

### Genetic Inactivation of *Emp2* Does Not Cause Nephrosis

**Figure 5:**
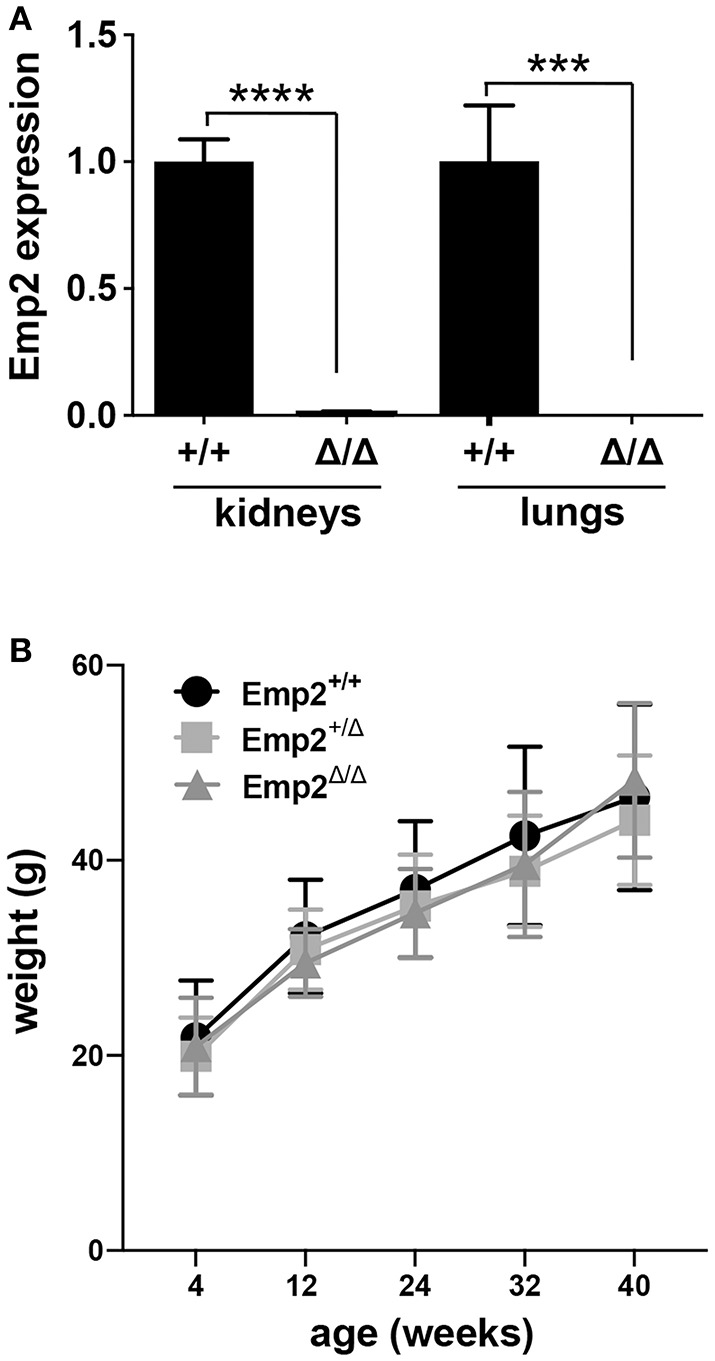
*Emp2* null mutant mice are viable. **(A)** Complete loss of *Emp2* expression in global *Emp2* null mutant mice. *Gapdh*-normalized *Emp2* transcript levels measured by qRT-PCR analysis showing total ablation of Emp2 expression in kidneys and lungs of homozygous *Emp2* null mutants (*Emp2*^Δ/Δ^). Mice > 4 weeks of age. *n* ≥ 3 animals per group. ***P < 0.0005, ****P < 0.0001. **(B)** Body weight is unaffected by the *Emp2* null allele. Comparable body weights between wild-type, heterozygous and homozygous *Emp2* null mutant mice (*Emp2*^+/+^, *Emp2*^Δ/+^, and *Emp2*^Δ/Δ^). *n* ≥ 3 animals per group.

An *Emp2* null mutant allele (*Emp2*^Δ^) was generated by crossing conditional floxed *Emp2* (*Emp2*^*flx*^) mice with *EIIa-Cre* transgenic mice (Supplementary Figure 1). Homozygous *Emp2* null mutants (*Emp2*^Δ/Δ^) were viable, live to adulthood, and are fertile. *Emp2*^Δ/Δ^ mutant animals have complete absence of *Emp2* expression in their lungs and kidneys by qRT-PCR analysis (Figure 5A). *Emp2*^Δ/Δ^ mutants had comparable body weights relative to their littermates control cohorts and lack any overt abnormality (Figure 5B).

**Figure 6:**
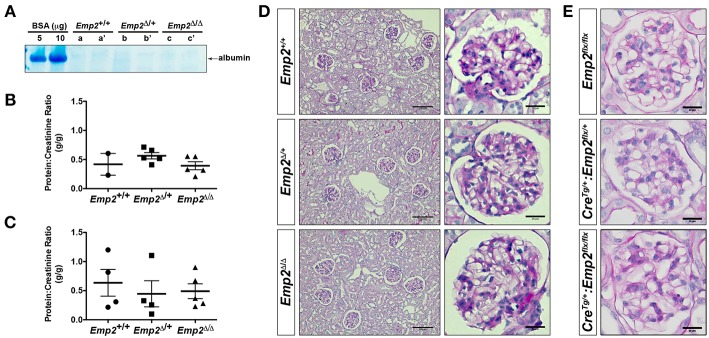
*Emp2* null mutant mice do not develop nephrosis. **(A)** Protein gel urinalysis do not show evidence of albuminuria in homozygous *Emp2* null mutant mice (*Emp2*^Δ/Δ^). Urine from 10-month-old animals run on SDS-PAGE alongside bovine serum albumin (BSA) standards and stained with Coomasie Brilliant Blue. **(B)** Urinary protein-creatinine ratios reveal lack of proteinuria in *Emp2* null mutants at 1-month of age. **(C)** No evidence of proteinuria at 10-months of age based on urinary protein-creatinine ratios. **(D)** *Emp2* null mutants have unremarkable overall renal and glomerular histology. **(E)** *Emp2* deletion in podocytes do not disrupt normal glomerular histology. Podocyte-specific *Emp2* conditional knockout (*Nphs1-Cre*^*Tg*+/^*:Emp2*^*flx*/*flx*^) mice have unremarkable and comparable glomerular histology relative to their wild-type control littermates (*Emp2*^*flx*/*flx*^ and *Nphs1-Cre*^*Tg*/+^*:Emp2*^*flx*/+^). Scale bars: 100 μm, D column 1; 20 μm D column 2 and E.

**Figure 7:**
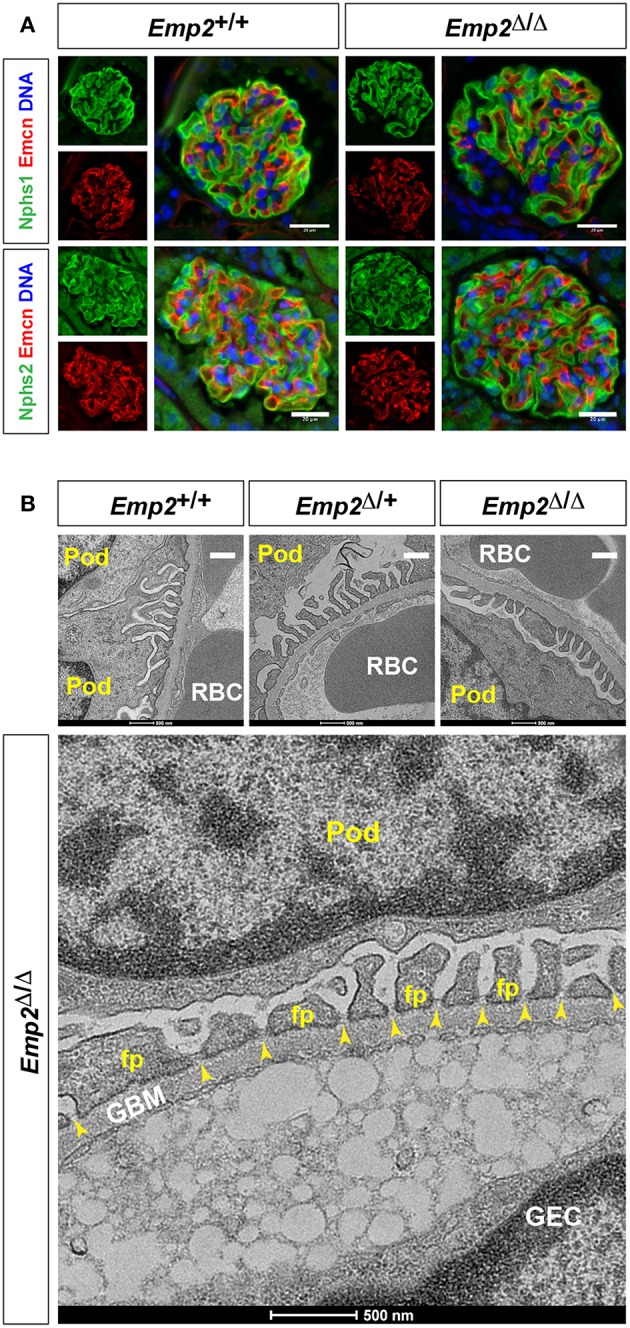
Slit diaphragms are intact in *Emp2* null mutants. **(A)** Comparable and regular expression of Nphs1 (nephrin) and Nphs2 (podocin) in wild-type control and homozygous *Emp2* null mutant (*Emp2*^Δ/Δ^) glomeruli. Immunofluorescence images of glomeruli stained for Emcn for the glomerular endothelium together with either Nphs1 or Nphs2. Scale bars: 20 μm. **(B)** *Emp2* null mutants have normal glomerular ultrastructure. Transmission electron micrographs of glomeruli showing normal organization, interdigitating podocyte foot processes and intact slit diaphragms. Pod, podocyte; GBM, glomerular basement membrane; GEC, glomerular endothelial cell; RBC, red blood cell; fp, podocyte foot process; and, yellow arrowheads, slit diaphragms. Scale bars: 500 nm.

Since mutations in the human *EMP2* gene have been previously linked to hereditary congenital nephrosis in a small subset of patients, we analyzed *Emp2* null mutant mice for signs of proteinuric kidney disease and glomerular defects. Mice at 10 months of age show no sign of proteinuria based on protein gel urinalysis (Figure 6A) and mice at 1 and 10 months of age show no sign of proteinuria base on urinary protein-creatinine ratios (Figures 6B,C). Gross renal and glomerular histology at 10 months of age also reveals normal overall kidney and glomerular morphology without any evidence of renal tubular protein casts or fibrosis in *Emp2* null mutant mice (Figure 6D). Gross pulmonary and renal arterial histology were unremarkable and comparable between control wild-type and *Emp2* null mutants even though *Emp2* is most prominently expressed in the lungs and vSMCs (Supplementary Figure 2). We also genetically inactivated *Emp2* exclusively in podocytes by crossing *Emp2*^*flx*^ mice with the podocyte-specific *Nphs1-Cre* deletor strain. Similarly, mice with targeted loss of *Emp2* in podocytes have normal overall renal histology and glomerular morphology (Figure 6E) and were non-proteinuric (not shown). Kidneys from *Emp2* null mutants have normal and regular expression of slit diaphragm proteins nephrin (Nphs1) and podocin (Nphs2) as seen by immunofluorescence (Figure 7A). Ultrastructure analysis by transmission electron microscopy shows that the structural integrity of the glomerular filtration barrier appears intact in *Emp2* null mutants with normal presence of interdigitating foot processes conjoined by slit diaphragms, unimpressive glomerular basement membrane (GBM), and fenestrated endothelial cells (Figure 7B).

Altogether, mutant mice aged up to 10 months were not visibly different from their littermate control cohorts and were non-proteinuric. At 10 months, renal histology and glomerular cytoarchitecture remain normal. These findings therefore indicate that *Emp2* is non-essential for normal glomerular development and function, and is consistent with lack of glomerular expression of *Emp2* in mice.

## Discussion

Steroid responsiveness is the most important prognostic factor in patients with nephrotic syndrome (29). As such, determining the underlying and potentially differing pathogenesis of SSNS in contrast to the known genetic variants of SRNS is of significant interest. Given the beneficial response of SSNS to immunosuppression such as steroids, potential immune mediated mechanisms of SSNS pathogenesis have been hypothesized with several risk loci being identified within the HLA-DQ and HLA-DR regions of the human leukocyte antigen (*HLA*) gene (4). Now a new Mendelian form of SSNS has been proposed with the discovery of biallelic mutations of *EMP2* in four individuals from three families with SSNS and SRNS (9). Homozygosity mapping and whole-exome sequencing initially identified a homozygous truncating variant in exon 4 of *EMP2* (Q62^*^) in a Turkish family with two siblings with SSNS. Further evaluation of a cohort of 1,600 individuals with NS revealed two additional individuals with biallelic mutations in *EMP2* demonstrating previously unreported missense mutations in exon 2 of *EMP2* (F7L and A10T). However, since this discovery of mutations in *EMP2* in these 4 individuals with NS, now tentatively classified as nephrotic syndrome type 10, evaluation for *EMP2* mutations in larger cohorts of nephrotic patients have not identified additional mutations or cases. In evaluation of a further 33 families with autosomal recessive SSNS, no evidence of pathogenic mutations of *EMP2* were found by Sanger sequencing (30). Additionally, in a whole-exome sequencing of 3,315 patients with chronic kidney disease, only two individuals were found to have heterozygous variants of in *EMP2* of unclear significance, and these mutations were deemed unlikely related to their underlying kidney disease (31). Whole exome sequencing of 363 unrelated family units with FSGS and 363 ancestry-matched controls also only detected suspicious variants of *EMP2* in control individuals (32). Altogether, these data suggest that pathogenic mutations in *EMP2* likely do not represent a significant portion of incident SSNS and may not be a monogenic etiology of proteinuric kidney disease.

Our data demonstrate that genetic deletion of *Emp2* does not cause proteinuric kidney disease in mice. Using quantitative real-time PCR analysis and histochemical characterization of *Emp2*^*lacZ*^ reporter mice, expression of *Emp2* is not enriched in the glomerulus or the podocyte as previously suggested (9, 28). Whole-mount β-gal histochemistry was used in lieu of antibodies against *Emp2* as commercially available antibodies gave only non-specific background signals by tissue immunostainings. The *Emp2*^*lacZ*^ reporter expression pattern we observed qualitatively correlates with relative mRNA expression in tissues based on qRT-PCR analysis, with most prominent expressions seen in lungs followed by the uterus by both methods. Furthermore, mice with global deletion of the *Emp2* gene were healthy, viable, and fertile, consistent with what has been reported independently by another group (26). Additionally, these mice did not manifest significant proteinuria by 10 months of age. Consistent with this, glomerular histology and cytoarchitecture are normal in mice with podocyte and global deletion of *Emp2*. *Emp2* null mutants show no evidence of podocyte effacement or perturbation of slit diaphragms. Together these data suggest that *Emp2* is non-essential for normal glomerular development and maintenance of integrity of the glomerular filtration barrier in mice. Our data, in addition to published reports of suspicious variants of *EMP2* in individuals without proteinuric kidney disease, suggest that mutations in *EMP2* may not be solely responsible for the development of early-onset childhood NS as previously reported (9). It is possible other modifying genes or environmental factors may account for the clinical heterogeneity seen in these cases.

Mouse *Emp2* protein has a strong homology with its human counterpart and shares a significant amino acid sequence identity (78%) (Supplementary Figures 3, 4), thereby justifying the use of a murine model to validate the hypothesis that loss of function of *EMP2* is causative for certain cases of SNSS. Additionally, two of the three reported missense mutations in *EMP2* (F7L and Q62^*^) occur in conserved amino acid residues between mice and humans further supporting the use of mice to model this condition (Supplementary Figure 3). However, the possibility remains that there are interspecies differences in expression and function of *EMP2*. And animal models of kidney disease do not always match the expected phenotype of human disease (33). Our study did not address these potential differences in *EMP2* expression and function in human tissues. Given that we did not observe glomerular expression of *Emp2*, we decided against examining the effect of *Emp2* deficiency in the context of a podocyte injury model. We suspect that a genetic cause of childhood onset NS would be less dependent on environmental injury for the development of the expected phenotype. Additionally, RNA-seq analysis of gene expression in mouse kidneys following unilateral ureteral obstruction injury reveals that *Emp2* expression was not particularly enriched in podocytes but rather expressed more significantly in proliferating proximal tubule and principal collecting duct epithelial cells (34).

Interestingly, we found that expression of *Emp2* was predominantly in arterial vascular structures across multiple tissues including the kidney. This finding is concordant to multiple recent studies that link *EMP2* to vascular function. *EMP2* has been previously linked to angiogenesis through both VEGF-dependent and VEGF-independent pathways in multiple tumor models (21, 23). In addition to tumorigenesis, alterations in *EMP2* expression has now been linked to failure of appropriate placental angiogenesis and pathogenic corneal neovascularization (26, 35). *EMP2* is currently being investigated as a novel target for cancer treatment and to reduce pathologic neovascularization (23, 24, 35–37). As such, it is essential to further define the role of *EMP2* in the vasculature as well as other tissues. In this study we found that expression of *Emp2* within the vasculature localized primarily to surrounding vSMCs. This is supported by a recent study implicating *Emp2* in a maladaptive pathway of vSMC proliferation in diabetic rats and raises the possibility of targeting *EMP2* for the treatment of vasculo-proliferative diseases in diabetes (38).

We also found expression of *Emp2* in multiple other smooth muscle tissues, including the esophagus, stomach, uterus and bladder, however, not in skeletal muscle tissues such as the thigh. Expression of *Emp2* was found in certain non-smooth muscle cells most prominently in the lung as well as the renal papilla. In the renal papilla, *Emp2* promoter activity co-localizes with calbindin (Calb1, a marker expressed in principal collecting duct epithelia), which is consistent with human and mouse RNA-seq data demonstrating prominent *Emp2* expression within the principal cells of the renal collecting duct (39, 40). While mice with loss of *Emp2* were viable and without overt pathology in tissues with high *Emp2* expression, such as the lung, it will be important to further define the role of *Emp2* in these tissues if future therapeutics targeting this pathway are to be developed.

Our data conclude that genetic deletion of *Emp2* does not cause proteinuric kidney disease in mice. Rather our findings further support a potential role of *Emp2* within the vasculature of multiple tissues including the kidney. Additional studies are warranted to further explore the specific role of *Emp2* in angiogenesis and vascular disease.

## Materials and Methods

### Mouse Strains and Husbandry

A floxed conditional allele of *Emp2* was created using a targeting vector obtained from the Knockout Mouse Project Repository (KOMP, https://www.komp.org). This vector incorporates a tandem bacterial *lacZ* (β-galactosidase) and neomycin-resistance selection (*neo*^*r*^) cassettes in between exons 1 and 2 of the mouse *Emp2* locus that are excisable with Flp recombinase. This vector also contains loxP (Cre recombinase recognition) sites flanking exons 2 and 3. DNA electroporation and clonal drug-selection of R1 (129X1 X 129S1) mouse embryonic stem (ES) cells were as described previously (41). Correctly targeted ES clones screened by Southern blot hybridization were used for blastocyst injection and generation of mouse chimera. Mouse lines were subsequently outbred onto a mixed genetic background. Mouse offspring harboring the targeted allele (*Emp2*^*lacZ*^) were used as reporter animals to assess *Emp2* promoter activity and tissue expression. A conditional floxed *Emp2* allele (*Emp2*^*flx*^) was generated by breeding *Emp2*^*lacZ*^ mice with Flp recombinase transgenic mice resulting in the removal of the *lacZ* and *neo*^*r*^ expression cassettes (42). Crossing of *Emp*^*flx*^ mice with the transgenic *EIIa-Cre [Tg(EIIa-cre)C5379Lmgd]* mouse line (43) results in germline inactivation of the *Emp2* locus and the establishment of first-generation heterozygous *Emp2* null mice (*Emp2*^Δ/+^). Mice with the genotype *Emp2*^Δ/Δ^ are classified as null mutants while those with genotypes *Emp2*^+/+^or *Emp2*^Δ/+^ are collectively classified as wild-type controls. Endothelial- and podocyte-specific knockout mice were derived from crosses between *Emp*^*flx*^ mice with the driver strains *Cdh5-CreERT2 [Tg(Cdh5-cre/ERT2)1Rha]* (44) and *Nphs1-Cre [Tg(Nphs1-cre)#Seq]* (45), respectively. Animals were genotyped by genomic PCR analysis using primers indicated in Supplementary Table 1. Mice were reared, bred, and characterized according to strict ethical guidelines approved by the Institutional Animal Care and Use Committee of Northwestern University.

### Gene Expression Analysis

Trizol reagent (Thermo Fisher Scientific, Waltham, MA) was used for extraction and purification of total RNA from isolated cells, tissues and organs. For isolation of glomeruli, anesthetized mice were systemically perfused with 0.3% (w/v) Fe_2_O_3_ particles in Hank's Balanced Buffer Solution (HBSS). Fe_2_O_3_-perfused kidneys were minced finely and digested with collagenase (10 mg/mL) at 37°C for 30 min. Dissociated kidney tissues were sieved through a 100-μm cell strainer, and the glomeruli captured using a magnet. Reverse transcription was completed using iScript cDNA synthesis kit (Bio-Rad, Hercules, CA) and real-time PCR was performed on an ABI 7500 thermocycler (Applied Biosystems, Foster City, CA) using iTaq Universal SYBR Green Supermix (Bio-Rad, Hercules, CA). DNA amplification threshold cycles values (C_t_) were used to evaluate gene transcript expression levels between genes of interests normalized to the housekeeping gene *Gapdh*. PCR primers used are listed on Supplementary Table 1.

### Histology and Histochemistry

Tissues and organs were routinely fixed in 4% formaldehyde in phosphate buffered saline (PBS, pH7.5) overnight at 4°C. Fixed tissues were embedded in paraffin blocks to produce 5-μm thick sections for routine histology (hematoxylin-eosin and periodic acid-aldehyde Schiff staining), and immunostainings. Standard methods for immunofluorescence and immunohistochemistry were carried out using antibodies listed in Supplementary Table 2. For *lacZ* reporter expression analysis, anesthetized mice with the *Emp2*^*lacZ*^ gene-trapped allele were perfusion-fixed with 300 μM chloroquine in PBS followed by 0.4% formaldehyde in PBS through the right cardiac ventricle. Subsequently, mice were perfused with lacZ rinse buffer (2 mM MgCl_2_, 0.01% sodium deoxycholate, 0.02% IGEPAL CA-630, and 300 μM chloroquine in PBS). Perfused and dissected organs were stained using an X-gal solution [1 g/L X-gal, 5 mM K_4_Fe(CN)_6_, and 5 mM K_3_Fe(CN)_6_ in lacZ rinse buffer] and incubated overnight at room temperature. Staining was quenched by incubation of tissues in phosphate buffered formalin solution.

### Urine Analysis

Urinary protein was measured using Bio-Rad Protein Assay (Bio-Rad, Hercules, CA) and urinary creatinine was analyzed through a microtiter-format colorimetric Jaffe reaction assay using alkaline picrate. Qualitative albuminuria was assessed on colloidal Coomasie dye-stained protein gels of urine samples alongside bovine serum albumin standards.

### Ultrastructure Analysis

Kidney specimens were fixed in 0.1 M cacodylate buffer (pH 7.5) containing 4% formaldehyde and 2% glutaraldehyde and post-fixed in 1% OsO_4_ prior to dehydration and embedding in epoxy resin for sectioning. Ultrathin resin sections were stained with uranyl acetate and lead acetate solution, and viewed in a FEI Tecnai G2 transmission electron microscope (ThermoFisher Scientific, Waltham, MA).

### Statistical Analyses

Quantitative data are shown as mean ± standard error of the mean (SEM). Statistical significance of quantitative results was evaluated using Student's *t*-test and ANOVA using GraphPad Prism version 8.00 (www.graphpad.com). *P* values of <0.05 were considered statistically significant.

## Data Availability

All datasets generated for this study are included in the manuscript/Supplementary Files.

## Author Contributions

MD, RS, TO, and SQ contributed to the design of the experiments. Experiments were performed by MD, RS, TO, AT, and UO. *Emp*2 mouse lines were developed by TO. MD, RS, TO, and SQ contributed to analysis of the data. The manuscript was written by MD, RS, and SQ. RS and SQ supervised the study. All authors contributed to the review and approval of the manuscript.

### Conflict of Interest Statement

The authors declare that the research was conducted in the absence of any commercial or financial relationships that could be construed as a potential conflict of interest.
