# Supplementary material for: Genetic Deletion of Emp2 Does Not Cause Proteinuric Kidney Disease in Mice
Source: Front Med (Lausanne). 2019 Aug 27;6:189. doi: 10.3389/fmed.2019.00189 (PMC6718710; doi:10.3389/fmed.2019.00189)
Supplement: Supplementary file 1 [file Data_Sheet_1.pdf]

Supplementary Material

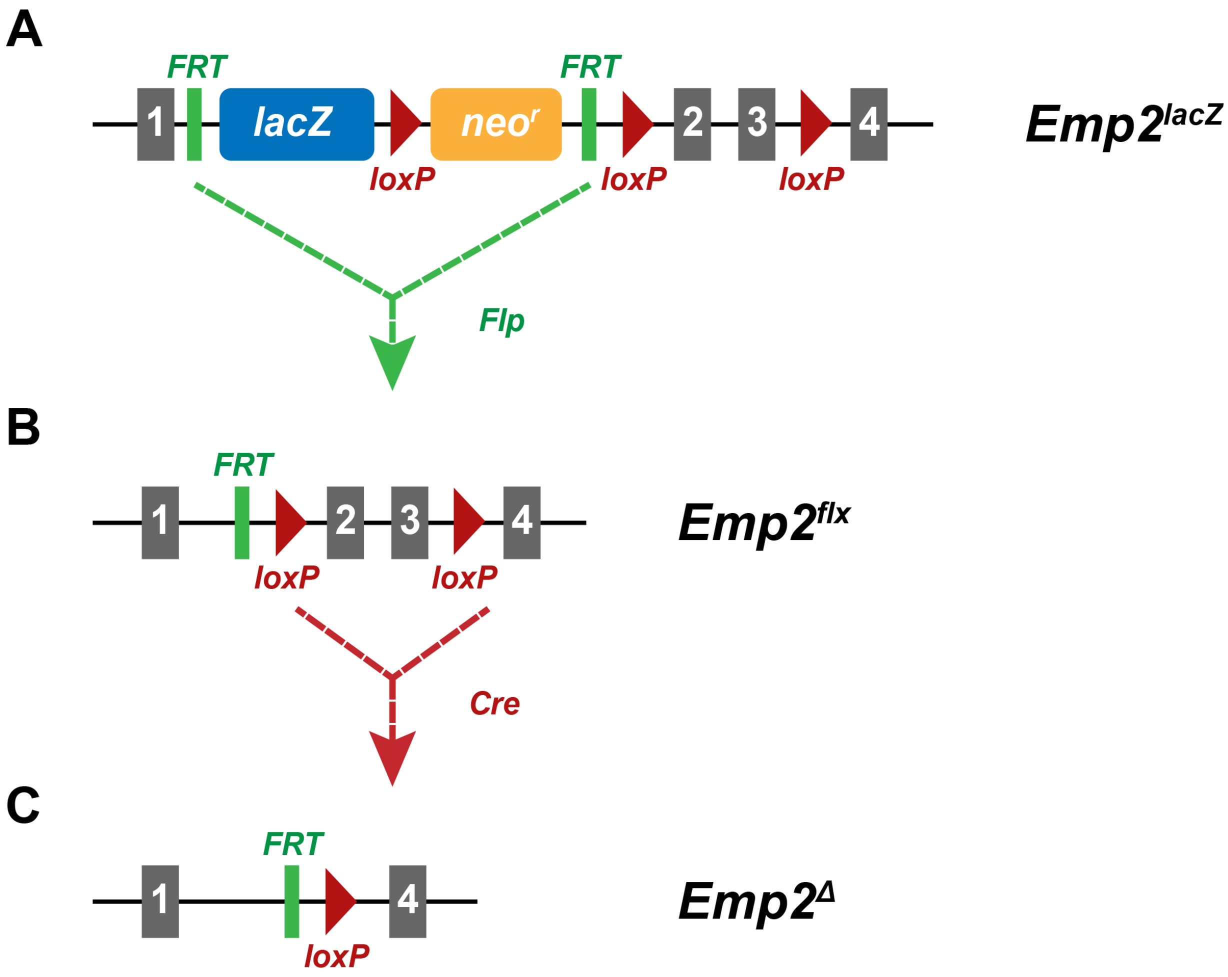

SUPPLEMENTARY FIGURE 1. Strategy for genetic inactivation of mouse *Emp2*. (A) Mice were developed using a targeting vector incorporating a bacterial *lacZ* and neomycin-resistance (*neo*^{r}) expression cassette between exons 1 and 2 and additional loxP sites flanking exons 2 and 3. **(B)** The *neo*^{r} cassette was removed by crossing mice carrying this targeted allele to a mouse carrying the FLP recombinase transgene generating floxed *Emp2* (*Emp2*^{flx}) mice. **(C)** *Emp2* null mutant allele (*Emp2*$^{\Delta}$) was generated by crossing *Emp2*^{flx} mice with various *Cre* transgenic mice resulting in excision of exons 2 and 3.

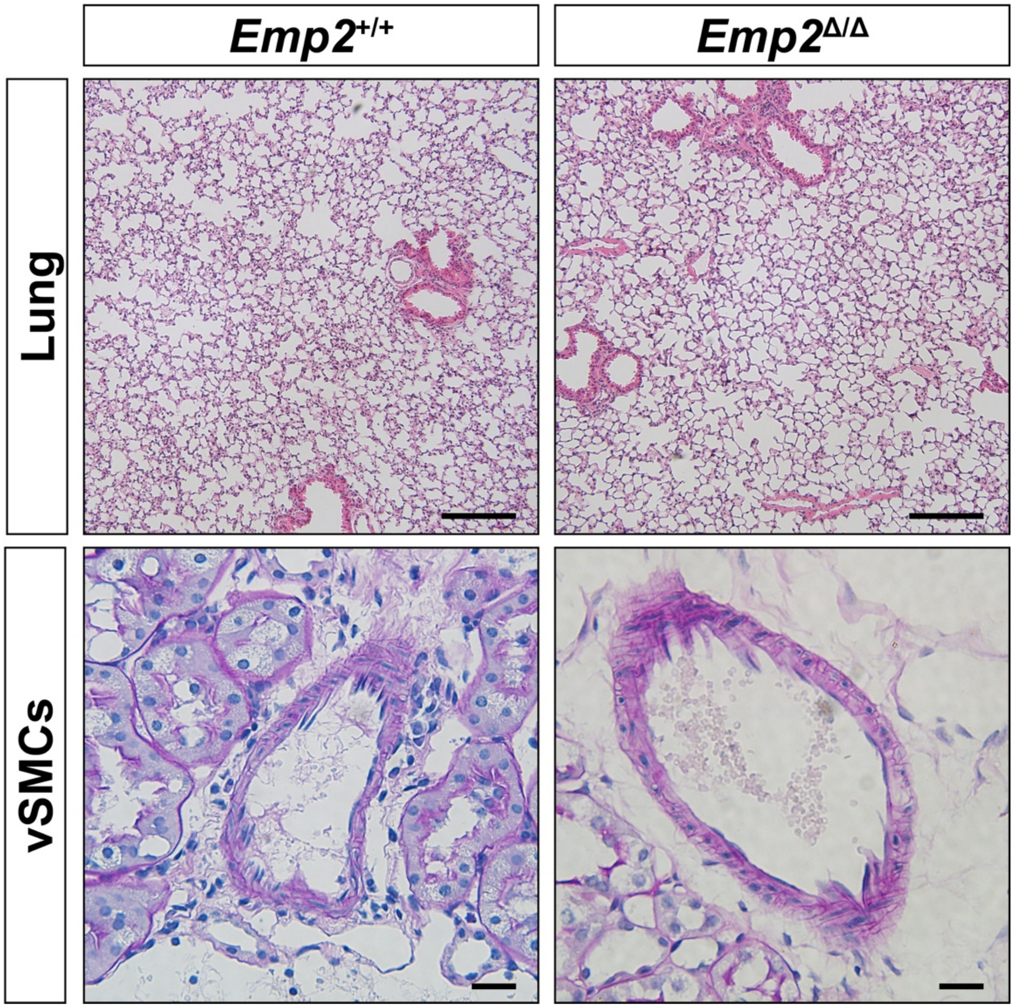

SUPPLEMENTARY FIGURE 2. Absence of overt pulmonary and vascular abnormalities in *Emp2* null mutant mice. *Emp2* null mutants have unremarkable lung and renal vascular histology at 10 months of age. Scale bars: 200 µm, lung images; 20 µm, vSMCs images.

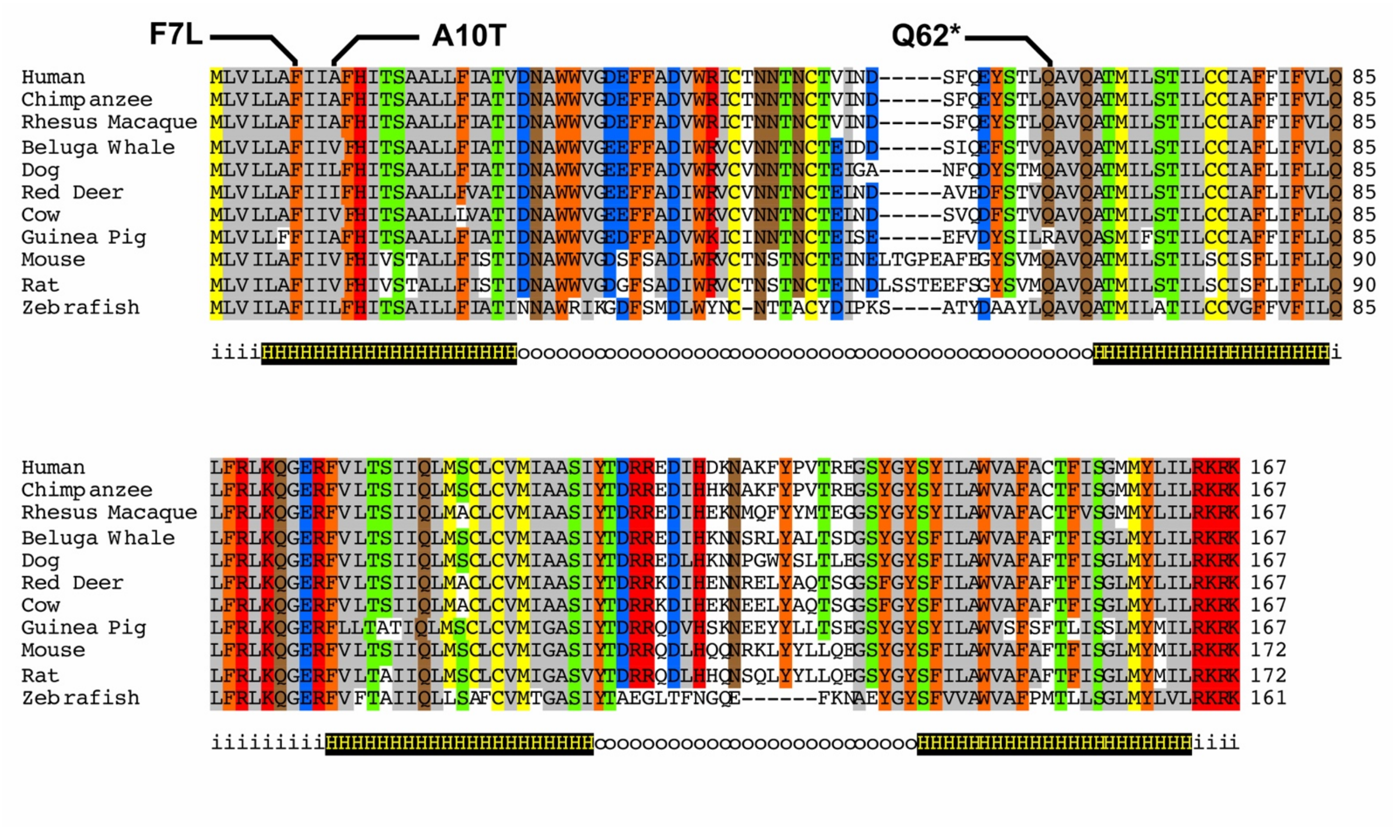

SUPPLEMENTARY FIGURE 3. Protein sequence alignment of *EMP2* orthologs. Clustal Omega (*https://www.ebi.ac.uk/Tools/msa/clustalo/*) protein sequence alignment of *EMP2* orthologs. Secondary structure predicted using JPred 4 (http://www.compbio.dundee.ac.uk/jpred/) is highlighted under the alignment (H, α-helix/transmembrane domain residue; i, internal loop residue; and, o, outside or external loop residue). Human *EMP2* mutations previously implicated in SSNS are indicated above the alignments.

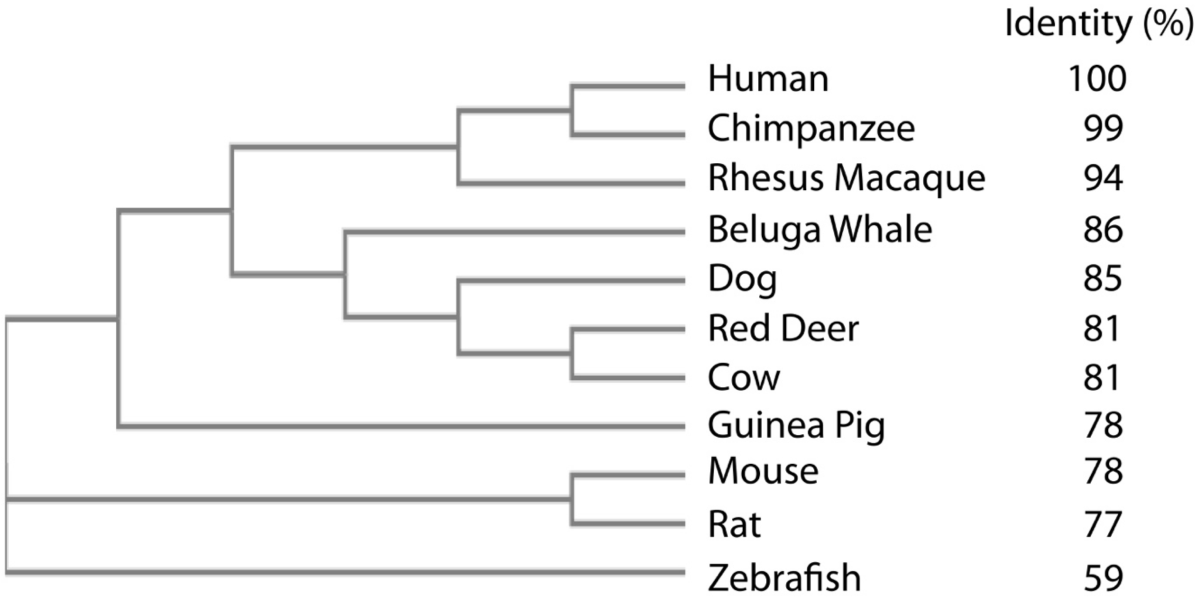

SUPPLEMENTARY FIGURE 4. *EMP2* phylogeny. Evolutionary relationship of different orthologs of *EMP2* showing percentage similarity in amino acid sequence identity.

SUPPLEMENTARY TABLE 1. List of PCR primers

Primer Name	Sequence (5’ → 3’)	Application
Emp2-Floxed-F1	GACAGTCTGCCAGAGCAAAG	genotyping
Emp2-Floxed-R1	CGTCCAAAGTGGTGAAGGAG	genotyping
Emp2-IVS1-F2	TTCAGTTATGTTCTATTTCCCACCT	genotyping
Emp2-IVS1-R1	TGACATCAAGGGACCAGGAG	genotyping
Emp2-En2SA-R1	GACCACCTCATCAGAAGCAG	genotyping
Emp2-F2	TTGAAGGTTATTCTGTGATGCAGG	qRT-PCR
Emp2-R2	AGCTGGATGATGGACGTCAG	qRT-PCR
Nphs1-qF	TATCGCCAAGCCTTCACAGG	qRT-PCR
Nphs1-qR	CAGCGAAGGTCATAGGGGTC	qRT-PCR
Gapdh-F	AAGGTCATCCCAGAGCTGAA	qRT-PCR
Gapdh-R	CTGCTTCACCACCTTCTTGA	qRT-PCR

SUPPLEMENTARY TABLE 2. List of antibodies

Antigen	IgG Species	Source*	Catalog Number	Application** (Dilution)
Calb1 (calbindin)	rabbit	MS	AB-1778	IHC (1:1000)
CD31 (Pecam1)	rat	TFS	MA5-13188	IF (1:100)
Des (desmin)	rat	Novocastra	NCL-c-DES-DERII	IHC (1:100)
Emcn (endomucin)	rat	Abcam	Ab106100	IF/IHC (1:250)
Nphs1 (nephrin)	goat	R&D	AF3159	IF (1:50)
Nphs2 (podocin)	rabbit	MS	P0372	IF (1:100)
PCK (pan-cytokeratins)	mouse	CST	4545	IHC (1:400)
Podxl (podocalyxin)	goat	R&D	AF1556	IF/IHC (1:100)
Tagln (transgelin/α-smooth muscle actin)	rabbit	Abcam	Ab14106	IHC (1:200)

*CST, Cell Signaling Technology (Danvers, MA; MS, Millipore-Sigma (Burlington, MA); R&D, R&D Systems (Minneapolis, MN); and, TFS, Thermo Fisher Scientific (Waltham, MA)

**IF, immunofluorescence; and IHC, immunohistochemistry
